# The Role of Platform Quality on Consumer Purchase Intention in the Context of Cross-Border E-Commerce: The Evidence from Africa

**DOI:** 10.3390/bs13050385

**Published:** 2023-05-06

**Authors:** Lintong Han, Yuehuan Ma, Prince Clement Addo, Miyan Liao, Jiaming Fang

**Affiliations:** 1 School of Management and Economics, University of Electronic Science and Technology of China, No. 2006, Xiyuan Ave, West Hi-Tech Zone, Chengdu 611731, China; 2 Faculty of Applied Sciences and Mathematical Education, Akenten Appiah-Menka University of Skills Training and Entrepreneurial Development, P.O. Box 1277, Kumasi 03220, Ghana

**Keywords:** cross-border e-commerce, information system success model, acculturation, African consumer

## Abstract

Africa, as one of the rapidly growing markets, presents a significant opportunity for cross-border e-commerce companies to penetrate their consumer market, which is in dire need of development. This study utilizes the Information System Success model to investigate the impact of cross-border e-commerce platform quality on consumers’ purchase intentions. Additionally, the study identifies the role of perceived value and trust in the purchase process. Moreover, the moderating effect of consumer acculturation on the relationship between cross-border platform quality and perceived value is examined. A total of 446 valid responses were obtained through a questionnaire survey and analyzed using structural equations. The findings reveal that platform information quality, system quality, and service quality significantly enhance consumers’ perceived value, thereby positively influencing their purchase intention. Furthermore, the results highlight the joint effect of perceived value and trust on purchase intention, and trust plays a mediating role in this relationship. The moderating effect of acculturation is also confirmed, indicating that it negatively moderates the impact of system and information quality on perceived value, while positively moderating the impact of service quality on perceived value. These findings complement and extend existing cross-border e-commerce research and provide valuable insights into the buying behavior of African consumers.

## 1. Introduction

The phenomenon of economic globalization has led to increased trade between countries worldwide, resulting in the rapid growth of cross-border e-commerce (CBEC) [1]. CBEC involves buyers and sellers from different countries or regions in contrast to traditional e-commerce. CBEC platforms should aim to establish an open and multidimensional multilateral economic and trade cooperation model, fully leverage the international market, and learn from each other’s experiences [2]. CBEC provides consumers with a more convenient and simplified shopping platform to purchase products from various countries or regions around the world without the need to leave their homes [3]. Industry analysis reports project the CBEC market to surpass $1 trillion by 2030 [4].

The extant research on CBEC has primarily focused on the burgeoning markets of China and EU countries, which are fast-growing countries for CBEC, while other regions have not received sufficient attention [5]. Despite being home to one of the most promising economies globally, the African continent remains relatively underdeveloped in this area [6]. Given the unique economic and cultural context of the African region, it stands to reason that African consumers’ behavior towards CBEC platforms may differ from that of consumers in other regions [7]. The antecedents of African consumers’ CBEC purchase intentions can provide companies with a more precise understanding of the African market’s needs and characteristics, thus offering valuable market intelligence. As such, this study represents a valuable contribution to the existing literature by addressing this gap and exploring this crucial issue.

The updated Information Systems Success (ISS) model has become prevalent in e-commerce as the industry has grown in scale [8,9]. Scholars have found that creating a high-quality website can increase users’ willingness to use e-commerce systems and their satisfaction, which can lead to market expansion and sales growth [10,11]. Nonetheless, CBEC exhibits distinct business models and market environments compared to traditional e-commerce [5], rendering it arduous to apply traditional e-commerce research findings to this context. Consequently, it is crucial to explore the influence of platform quality on consumers’ purchase intention in the CBEC setting.

In recent years, consumer acculturation has been demonstrated to significantly impact various behaviors such as shopping, dining, and traveling, as evidenced by research studies [12,13,14]. Scholars have found that creating a high-quality website can increase users’ willingness to use e-commerce systems and satisfaction, which can lead to market expansion and sales growth [15,16,17]. However, consumers are increasingly exposed to cross-regional consumer cultures during cross-border transactions [18]. Therefore, it is deemed imperative to investigate the moderating influence of consumer acculturation in CBEC settings.

To our knowledge, this study is the first to explore the behavior of African consumers of CBEC and to evaluate the impact of CBEC platform quality on consumers’ purchase intentions. This study aims to answer two fundamental questions: (1) What is the impact of the quality of the CBEC platform, including information quality, system quality, and system quality, on the purchase intention of African consumers? and (2) How does acculturation moderate the effect of platform quality on African consumers’ purchase intentions? To address these questions, the study employs structural equation modeling to assess the influence of CBEC platform information quality, system quality, and service quality on African consumers’ purchase intentions, based on the ISS model. The study also investigates the role of trust and especially perceived value in the process. Furthermore, the study introduces the construct of acculturation to examine the impact of platform quality on consumers’ behavioral intentions while further exploring the moderating role of consumer acculturation on the relationship between CBEC platform quality and consumers’ perceived value. This study not only contributes to the existing ISS model and acculturation research but also provides a theoretical explanation for African consumer behavior in the CBEC context. The findings may also be useful for merchants in developing effective platform management and marketing strategies in practice.

## 2. Literature Review and Theory Development

### 2.1. Cross-Border E-Commerce

Cross-border e-commerce (CBEC) refers to the process of online trade dealings between trading entities in different countries through e-commerce platforms [1,19]. Compared with traditional e-commerce, CBEC is typically characterized by the realization of cross-country online trade [20], which facilitates international trade for enterprises of different regions and scales [1]. At present, global CBEC development is increasingly positive [21].

In recent years, most studies on CBEC have focused on three topics: logistic issues, consumer behaviors, and CBEC policies. Cross-border logistics play a very important role in the process of CBEC [22]. Prior studies of logistic issues primarily cover logistics distribution networks [23,24], overseas warehouses [25,26], cross-border payments [27], and customs clearances [28]. Consumer behavior is a focused topic in CBEC, covering such areas as consumer trust [29], perceived value, and purchase intention [30,31,32]. Moreover, supportive and legal policies are of great concern in promoting the CBEC market [33,34].

At present, existing CBEC research focuses mainly on China and EU countries, where CBEC is growing rapidly [5,35,36]. However, there is a lack of research on the behavior of African consumers in the context of CBEC. In recent years, the e-commerce user community in Africa has been expanding. In particular, the implementation of the African Continental Free Trade Area (AfCFTA) agreement has lowered barriers to cross-border payment and transport clearance, it has provided great convenience for African consumers to purchase on CBEC platforms [37]. Alibaba, Jumia, and Takealot have established a dominant presence in the African e-commerce market. For instance, Alibaba’s CBEC arm, AliExpress, has successfully served 4.2 million customers since its launch in Africa. Furthermore, the burgeoning e-commerce market in Africa has piqued Amazon’s interest, leading the tech giant to expand its operations on the continent with plans to launch its e-commerce platform in Nigeria and South Africa in 2023. These examples demonstrate the significant growth and potential of CBEC in Africa, which is emerging as a key driving force behind the AfCFTA [38]. Thus, this paper takes the Africa CBEC market as the research background to explore the influence of the ISS model on African consumer purchase behavior.

### 2.2. Information System Success Model

The Information Systems Success model (ISS) was initially introduced by DeLone and McLean in 1992, positing that the success of an information system can be gauged by the system quality and information quality [39]. Subsequently, DeLone and McLean [10] refined the ISS model by augmenting the user evaluation of information systems into three dimensions, namely information quality, system quality, and service quality. These dimensions enable a more comprehensive appraisal of the effectiveness of information systems.

The enhanced ISS model has gained widespread use in various domains to evaluate the impact of information, system, and service quality on user satisfaction, usage, trust, perception, and operational efficiency. Notably, studies applying the improved ISS model in the realms of online learning, smart cities, and mobile banking have demonstrated that the quality of information system profoundly affects user satisfaction and intention to use [40,41,42,43,44]. Furthermore, the system quality and service quality of digital libraries are critical factors in shaping users’ perception of usefulness [45], while system quality and information quality are the key drivers of the perceived usefulness of e-learning systems [46]. Additionally, the quality of mobile commerce has a substantial positive correlation with users’ trust [47], and e-government systems’ quality positively impacts operational efficiency [48].

Although the initial application of the ISS model was in the domain of traditional information systems, its utilization in e-commerce has become prevalent with the rapid development of the latter [8,9,11,49]. However, despite economic globalization leading to the emergence of CBEC, there has been limited exploration of how quality elements of CBEC impact consumers’ perceptions and purchase intentions in this context [19]. In this study, we aim to contribute to this literature stream by examining the behavior of African buyers in the context of CBEC using the ISS model. Specifically, we investigate the potential relationships between three quality indicators—system quality, service quality, and information quality of CBEC platforms—and buyers’ perceptions and behaviors.

### 2.3. Consumer Acculturation

Acculturation can be defined as the alteration of an individual’s preferences following exposure to people or groups from different cultural backgrounds [50]. This process may also be explained as a consequence of cross-cultural contact, or changes in the behavior or psychology of individuals (or groups) that arise from their interaction with different cultures [51]. Researchers have come to realize that acculturation can occur not only between national and host cultures but also between local and global consumer cultures [52], thereby prompting widespread investigation of consumer acculturation.

Prior research suggests that varying degrees of acculturation can significantly affect consumer behavior in several domains, such as shopping, traveling, and dining [12,13,14]. For instance, Western acculturation behaviors exhibited by consumers may drive their purchase intentions [12], and consumers’ level of acculturation can significantly influence their motivation, attitudes, and behaviors while traveling [14]. Additionally, immigrant consumers’ food and restaurant preferences are closely linked to their degree of acculturation [13]. Conversely, in the online sphere, consumer acculturation research has primarily focused on the social media scene. For example, Kizgin [15] explored how acculturation resulting from interactions between immigrant consumers and others on social media affects purchase intentions. Furthermore, acculturation can also influence consumers’ attitudes and willingness to engage with electronic word-of-mouth [17].

Existing research has indicated that acculturation can influence consumers’ attitudes toward e-commerce acceptance in the context of online shopping [16,53]. When it comes to CBEC, consumers are frequently exposed to cultures from different regions as they engage in the purchase of cross-border products and services [18]. Therefore, differences in acculturation may lead to variations in consumer behavior. Despite the significance of this issue, little exploration has been conducted. Our study contributes to this area of research by shedding light on the moderating influence of acculturation.

## 3. Research Model and Hypotheses

### 3.1. Information Quality and Perceived Value

The concept of information quality is concerned with the degree to which users can adapt to the characteristics of information presented on a website [54]. In the present study, the construct of information quality is operationalized in terms of accuracy, usefulness, trustworthiness, and adequacy of information provided by CBEC platforms [11,55]. Firstly, the detailed and precise product information supplied by the CBEC platform helps cross-border consumers to comprehend the product more effectively, enabling them to make well-informed and rational purchase decisions to satisfy their utilitarian motives, thereby increasing the perceived value [56,57]. Next, consumers have a relatively single channel to obtain relevant information and usually rely on the information provided by the platform while purchasing goods on CBEC platforms. The richer the information that the platform offers to users about the product or service, the less time and effort they will expend in comprehending and utilizing the platform, resulting in reduced perceived costs to the consumer, thus contributing to the overall perceived value [58]. Moreover, when the platform displays tailored and specific information that caters to consumers’ needs, it can offer customers quick and convenient services with personalized features, thereby enhancing consumers’ hedonic value and contributing to the increase in perceived value [56,59]. Based on these propositions, we posit the following hypothesis:

**H1.**  *The information quality of the CBEC platform is positively correlated with consumers’ perceived value.*

### 3.2. System Quality and Perceived Value

System quality can be defined as the technical and functional performance that an information system aims to achieve [11,60]. Key indicators of system quality include usability, reliability, adaptability, and response time [11,61]. Previous research has demonstrated that consumers are able to quickly adapt to using a platform if it offers a clear layout and simple operation, leading to increased comfort and confidence [62]. Additionally, a platform’s aesthetic design and rich functionality provide hedonic value to consumers [63], which can effectively enhance their perceived value. However, encountering system instability or slow response time during platform usage can lead to increased search and organization costs, as well as negative shopping experiences, ultimately weakening perceived value [64]. Consumers using CBEC platforms are usually relatively unfamiliar with the platform’s systems and are thus more sensitive to the perception of system quality [19]. Therefore, this study argues that the above role of e-commerce platforms is more applicable in CBEC platforms, and thus proposes:

**H2.**  *The system quality of the CBEC platform is positively correlated with consumers’ perceived value.*

### 3.3. Service Quality and Perceived Value

In the context of this study, service quality indicates the service environment, product quality, and other ancillary services provided by CBEC platforms and merchants for consumer shopping [65]. Previous research shows that service quality has a positive relationship with consumer perceived value [66,67,68]. First of all, the service quality provided by platforms and merchants can have an effect on perceived value by influencing consumers’ emotions [67]. The better the service environment, product quality, and service guarantees of the platform in terms of service environment and products, the higher the likelihood of positive consumer emotions [69], and positive emotions contribute to a high level of perceived value [70,71]. In addition, the evaluation of consumers’ perceived value is directly influenced by the external service environment and product quality, CBEC platforms involve additional complexities such as language barriers, different payment methods, and shipping logistics, which can make the customer experience more challenging. The high service quality in all dimensions provided by the platform to consumers also contributes to their perceived value [72]. These ideas are summarized with the following hypothesis:

**H3.**  *The service quality of the CBEC platform is positively correlated with consumers’ perceived value.*

### 3.4. Moderating Role of Acculturation

Consumer values are shaped by the extent consumers adapt to a particular culture, including their needs, expectations of products, and criteria for evaluation, which ultimately affect their selection and utilization of goods and services [73,74]. In the context of CBEC platforms in the global marketplace, merchants and consumers often come from diverse geographical and cultural backgrounds [75]. The acculturation demonstrated by consumers can lead to variations in their attitudes and behaviors related to the acquisition and utilization of goods and services [76].

In the context of e-commerce platforms, Lv et al. [77] highlight that sellers typically possess more comprehensive product information than consumers, making it necessary for consumers to rely on sellers to make informed purchasing decisions. However, Chai and Dibb [78] suggested that more acculturated consumers may possess a higher degree of familiarity with both product information and market conditions, allowing them to evaluate products independently. Additionally, such consumers tend to compare information across multiple channels and may evaluate the information provided by the CBEC platform with a higher degree of scrutiny, thus enabling them to identify and correct misinformation or misleading information [73]. As a result, highly acculturated consumers are more likely to rely on their own knowledge and judgment, rather than solely depending on the quality of the information provided by CBEC platforms to enhance their perceived value. Therefore, we propose the hypothesis:

**H4.**  *Acculturation has a negative moderating effect on the relationship between information quality and consumers’ perceived value in CBEC platforms.*

In CBEC platforms, consumers with a high level of acculturation are more likely to familiarize themselves with the platform’s rules, processes, and services, leading to a greater focus on product quality, price, and after-sales service rather than on technical indicators such as the platform’s system stability and speed [79]. Consequently, high acculturation consumers tend to have relatively low requirements for platform system quality, resulting in a less pronounced impact of system quality on their perceived value. However, if CBEC platforms encounter system quality issues, high acculturation consumers may become dissatisfied, negatively affecting their perceived value [64]. Nevertheless, these consumers tend to be more risk-averse and can effectively respond to mitigate risks when encountering such problems [80]. Therefore, the effect of system quality on consumers’ perceived value is weakened by high acculturation. As a result, we propose the following hypothesis:

**H5.**  *Acculturation has a negative moderating effect on the relationship between system quality and consumers’ perceived value in CBEC platforms.*

In CBEC, the cultural differences between consumers and merchants have a significant influence on consumers’ shopping experiences. The ability of consumers to adapt to cross-cultural service approaches and product features affects their level of pleasure and satisfaction and reduces the stress associated with cross-cultural platforms [16]. A consumer’s evaluation of e-commerce service quality is influenced by their cultural background and cognitive system. High acculturation consumers are more likely to appreciate the value of attentive and differentiated services provided by the platform [81]. Consequently, they are more likely to assess the impact of e-commerce platform service quality on their personal experience when conducting CBEC transactions. Therefore, the following hypothesis is proposed:

**H6.**  *Acculturation has a positive moderating effect on the relationship between service quality and consumers’ perceived value in CBEC platforms.*

### 3.5. Perceived Value and Purchase Intention

Purchase intention refers to the consumer’s attitude and willingness to purchase a product [82]. Existing research shows that the perceived value of a product or service experience to consumers has a significant impact on their purchase decisions and intentions [83,84]. On the one hand, consumer perceived value influences purchase intentions by increasing their preferences for a particular merchant, brand, or product [85,86]. In the context of e-commerce research, consumer preferences represent their liking for merchants and products [86], and high perceived value significantly increases consumer preferences [87], which in turn enhances consumers’ intentions to purchase products. On the other hand, previous studies have shown that consumer perceived value and satisfaction have a facilitating effect on purchase behavior [88,89]. Consumers with high perceived value tend to have higher satisfaction with merchants and products [90], which in turn increase consumers’ attitudes and propensity to purchase or recommend products [89]. These ideas are summarized with the following hypothesis:

**H7.**  *The consumers’ perceived value is positively correlated with consumers’ purchase intention.*

### 3.6. Perceived Value and Consumer Trust

Trust refers to the credibility that one party has in the other party’s ability to deliver on the promise of cooperation [91]. In this study, trust indicates the extent to which consumers recognize the reputation of CBEC platforms, merchant service capabilities, and product quality. Previous studies have shown that the consumer’s perceived value has a positive impact on trust [92,93]. First, high perceived value can be seen as the positive added value that a product possesses [94], and high added value increases the product’s recognition in the minds of consumers during e-commerce transactions [93], which in turn enhances consumer trust. Second, the higher the perceived value of consumers when shopping on CBEC platforms, the more credible they perceive the platform-related subjects to be as well [55], which promotes increased consumer trust. These ideas are summarized with the following hypothesis:

**H8.**  *The consumers’ perceived value is positively correlated with consumer trust.*

### 3.7. Consumer Trust and Purchase Intention

In an online shopping environment, consumer trust helps to reduce uncertainty when conducting product transactions [95]. Existing research suggests that consumer trust has a positive effect on purchase intention [92,93,95]. First of all, high levels of trust can significantly reduce consumers’ behavioral hesitation during e-commerce shopping [96,97]. The higher the level of trust consumers have in the merchant and the product, the greater their propensity to purchase the product [98], which can facilitate consumers’ purchase behavior. In addition, the development of consumers’ trust in products and merchants increases their satisfaction when shopping [92,93], which contributes to the growth of consumers’ purchase intention and facilitates their purchase behavior. These ideas are summarized with the following hypothesis:

**H9.**  *Consumer trust is positively correlated with consumers’ purchase intention.*

**Figure 1:**
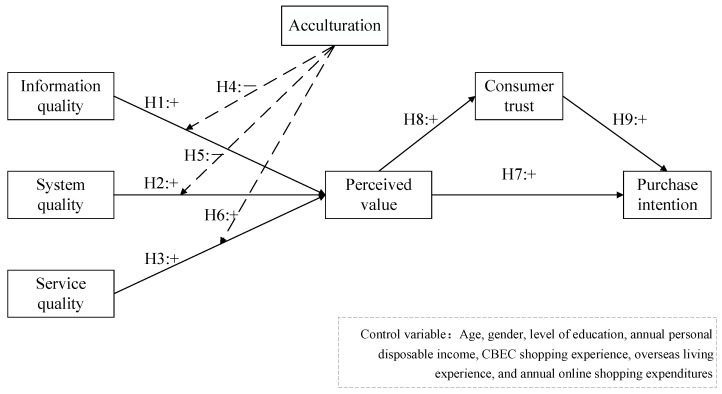
Research model.

Based on the above hypotheses, the research model is shown in Figure 1.

## 4. Research Methodology

### 4.1. Sample and Data Collection

The present study employed the questionnaire method as a means of data collection to investigate the relationship between CBEC platform quality and African consumers’ purchase intention. The questionnaire comprised four main sections. The introductory section provided a brief overview of the study and instructions for completing the questionnaire. The second section included demographic questions that covered respondents’ age, gender, level of education, annual personal disposable income, CBEC shopping experience, overseas living experience, and annual online shopping expenditures. In the third section, we defined cross-border e-commerce platforms and listed examples of CBEC platforms, such as Amazon, eBay, and Alibaba. This guided respondents to recall their most recent shopping experience through CBEC platforms. Additionally, respondents were asked to provide the name of the CBEC platform they had most recently shopped on, and we screened the questionnaire to retain respondents from larger platforms, thereby avoiding significant quality differences in the CBEC platforms chosen by respondents. The fourth and final section investigated respondents’ perceptions of the quality of CBEC platforms in terms of information quality, system quality, and service quality. Additionally, respondents’ perceived value, trust, and purchase intention of the CBEC platform and the degree of respondent acculturation were also assessed. To ensure the accuracy and impartiality of the questionnaire content, a pretest was conducted with 102 individuals, and the questionnaire was revised based on their feedback. The study participants were selected from university students who are more proficient and widely use CBEC platforms in Africa.

In February 2023, the formal survey was conducted to investigate African consumer behavior on CBEC platforms. University students were randomly recruited offline, and participation in the survey was voluntary. Out of the 545 questionnaires collected, 446 questionnaires were deemed valid after eliminating those that did not purchase products from CBEC platforms, answered all questions the same way, and took less than 180 s to complete. The valid questionnaire rate was calculated to be 76.33%. The study found no significant non-response bias in the sample, as determined through a series of χ^2^ tests. No major demographic variables were found to be significantly different between early and late-stage respondents. The sample statistics are presented in Table 1.

We retained participants with varying ages and education levels to ensure sample diversity, despite their low proportion as shown in Table 1. To test the robustness of our results, we conducted a separate analysis without these participants and found no significant difference from our original findings. The gender imbalance observed in our sample may be linked to persistent gender inequalities in African countries, where men tend to have an advantage in education and consumption [99,100].

### 4.2. Measurement of Variables

Seven additional demographic variables were included in the model as control variables to control for potential spurious regression effects. These variables include age, gender, level of education, annual personal disposable income, CBEC shopping experience, overseas living experience, and annual online shopping expenditures. We chose these variables based on previous studies that have shown they can have an influential effect on consumer purchase behavior. Age and gender are important factors that can affect consumer behavior, as different age and gender groups may have different preferences and attitudes towards online shopping. Education level and personal disposable income are also relevant factors as they can influence consumers’ purchasing power and decision-making processes. Moreover, CBEC shopping experience, overseas living experience, and annual online shopping expenditures are included as they reflect consumers’ familiarity with cross-border e-commerce platforms and their online shopping behavior. By controlling for these variables, we can isolate the effect of the perceived quality factors on consumer satisfaction and purchase intention.

All constructs in the questionnaire, except for the control variables, were evaluated on a seven-point Likert scale (“1” for “completely disagree” and “7” for “completely agree”). All seven constructs were adapted from mainstream international journal papers and included at least three items. All of the construct measures and their literature sources are included in Table 2.

### 4.3. Reliability and Validity

The reliability, convergent validity, and discriminant validity of the theoretical model scales were examined by using SmartPLS 3. The composite reliability (CR) coefficients of all latent variables are greater than 0.8 and the Cronbach’s Alpha coefficients are greater than 0.7, which indicated that the reliability of the scale is high and has good internal consistency. The average variance extracted (AVE) values are higher than 0.5, indicating that the scale has good convergent validity, as shown in Table 3. All the AVE are higher than 0.5, indicating that the latent variables have good discriminant validity among themselves, as shown in Table 4.

### 4.4. Common Method Bias

There is evidence that common method bias may be a problem when self-reported questionnaires are used as the data collection method may adversely affect the reliability and validity estimates of the scales and the estimation of the relationship parameters between the variables. In particular, the data are vulnerable to common method bias when participants answer questions in a single questionnaire at a given point in time. Given this study used a questionnaire method to collect data from the same source, it was necessary to verify whether the assessment results were affected by common method bias. For this purpose, a Harman single-factor test was conducted. The results of the analysis showed that the first factor explained only 18.866% of the total variance, which is much less than the critical value of 40%. This indicates that the common method bias is not serious in this study.

## 5. Data Analysis and Results

**Figure 2:**
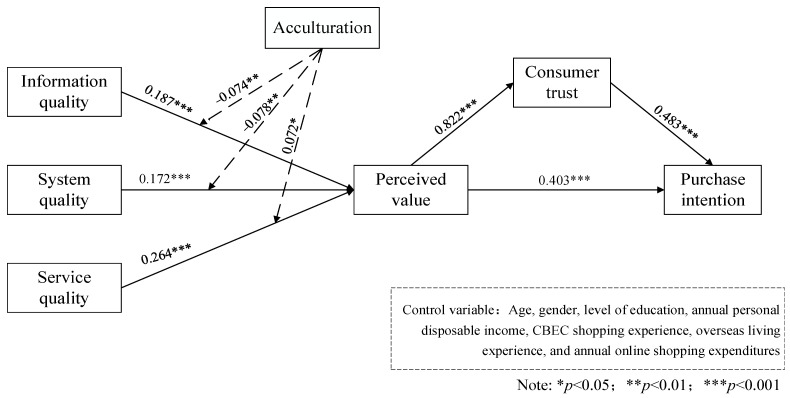
Model test results.

We used the partial least squares path model (PLS-PM) approach to test the appropriateness of the structural model using the PLS algorithm provided by SmartPLS 3 [105]. Significance tests for the parameters in the model were performed by the 500-times PLS Bootstrapping method. The specific normalized path coefficients are shown in Figure 2.

Hypotheses H1, H2, and H3 propose that information quality, system quality, and service quality, respectively, have positive effects on consumers’ perceived value. The empirical results indicate that all information quality (β = 0.187, *p* < 0.01), system quality (β = 0.172, *p* < 0.01), and service quality (β = 0.264, *p* < 0.01) have significant positive effects on perceived value, providing support for H1, H2, and H3.

Hypotheses H4 and H5 suggest that acculturation can weaken the influence of information quality and system quality of CBEC platforms on consumers’ perceived value. The results of the hypothesis tests show that the effects of acculturation on both information quality and consumer perceived value (β = −0.074, *p* < 0.05), and system quality and consumer perceived value (β = −0.078, *p* < 0.05) are negatively significant. Therefore, hypotheses H4 and H5 are supported. Hypothesis H6 argues that the degree of acculturation enhances the impact of CBEC platform service quality on consumers’ perceived value. The results of the hypothesis test showed that the interaction effect of acculturation and service quality has a significant positive effect on perceived value (β = 0.072, *p* < 0.10). It indicates that the higher the degree of acculturation, the stronger the effect of service quality on consumers’ perceived value. Therefore, hypothesis H6 was supported.

Hypothesis H7 examines the effect of consumer perceived value on consumer purchase intention. The results of the hypothesis test showed that when consumers’ perceived value is high, their purchase intention is also significantly strengthened (β = 0.403, *p* < 0.01). Therefore, hypothesis H7 is supported. In addition, consumers’ perceived value can significantly enhance consumers’ trust in CBEC platforms (β = 0.822, *p* < 0.01), and high trust in e-commerce platforms can significantly enhance consumers’ intention to purchase (β = 0.483, *p* < 0.01). That is, hypothesis H9 was supported. Further test of mediating effect indicated a mediating role for consumer trust in the effect of perceived value on purchase intention (β = 0.397, 500 bootstraps 95% CI = [0.305, 0.494]).

## 6. Discussion and Implications

### 6.1. Discussion

The purpose of this study is to examine the effects of information quality, system quality, and service quality on the purchase intention of African consumers in the CBEC environment, using the ISS model. Moreover, this investigation is conducted from the perspective of CBEC platform quality, focusing on the role of perceived value and trust in shaping purchase intention. To achieve these objectives, the study employs empirical analysis, which reveals that the information quality, system quality, and service quality of CBEC platforms all have a positive impact on consumers’ perceived intention to purchase.

As CBEC platforms and their associated merchants or products continue to improve their information, system, and service quality, consumers’ perceived value is expected to become increasingly important. By comparing the effectiveness of information quality, system quality, and service quality, it is observed that service quality has a much greater impact on consumers’ perceived value than the other two factors. Therefore, while consumers’ perceived value in the CBEC shopping process is determined by the joint effect of all three qualities, high service quality plays a dominant role in enhancing consumers’ perceived value.

Furthermore, the study finds that consumers’ purchase intention is strongly influenced by their trust and perceived value of the CBEC platform. Specifically, perceived value has a direct impact on purchase intention, while trust mediates the relationship between perceived value and purchase intention.

The research also investigates the moderating effect of consumers’ degree of acculturation on the influence of platform quality on purchase intention in the CBEC context, to explore the underlying mechanism of this relationship. The results show that acculturation can weaken the impact of CBEC platform information quality and system quality on consumers’ perceived value and enhance the impact of the service quality.

### 6.2. Theoretical and Practical Implications

This study contributes to the existing literature on CBEC by extending the scope of research beyond China and EU countries, which have been the primary focus in previous studies [5]. While Africa represents a rapidly growing market for CBEC, it has received limited attention thus far [6]. This study addresses this gap by exploring the purchasing behavior of African consumers in CBEC.

Moreover, this study extends the ISS model by investigating the quality of CBEC platforms. Previous research has primarily applied the ISS model in the context of traditional e-commerce [8,9], with limited attention given to CBEC. However, buyers and sellers in CBEC usually come from different countries or regions, and their business models and market environments also differ significantly from those of traditional e-commerce [5], so the findings of traditional e-commerce studies are difficult to apply in the CBEC context. This study fills this gap by examining the impact of quality elements on consumers’ perceptions and purchase intentions and emphasizing the importance of perceived value and trust. Additionally, this study reveals the mediating and moderating mechanisms underlying the relationship between quality elements and consumer behavior in CBEC.

Finally, this study contributes to the field of consumer acculturation research by exploring the moderating role of consumer acculturation in CBEC platforms. Previous research has focused on consumer offline behavior, social media behavior, and traditional e-commerce behavior [12,15,16,17]. Consumers in CBEC contexts are exposed to cross-regional consumer cultures [18], which necessitates a clarification of the role of consumer acculturation. This study expands the application scenarios of consumer acculturation, highlighting its importance in CBEC.

The findings of this study have practical implications for CBEC platforms that aim to expand their market into Africa. The key to increasing trust and purchase intention is by improving consumer perceived value. This can be achieved by enhancing the quality of the platform’s system, information, and service. For instance, the CBEC platform should provide accurate, detailed, and timely information on product descriptions, prices, and pictures. It can also ensure stability, efficiency, and ease of use in its system, taking into account the local communication infrastructure in Africa, thereby enhancing the African consumer’s shopping experience. Pre-sales and after-sales services that are professional, friendly, and attentive can provide comprehensive consultation and support for consumers, especially in the relatively weak logistics link service levels in Africa, which should be strengthened. Another important factor to consider is the impact of acculturation. Research indicates that cultural adaptation moderates the effect of CBEC platform quality on consumers’ perceived value, and this effect varies across information and system quality and service quality. Therefore, CBEC platforms need to be mindful of the impact of acculturation to better cater to consumers’ differentiated needs. For example, low acculturation consumers are more sensitive to negative effects arising from information and system quality, hence the platform should avoid unstable systems and misleading information. Additionally, platforms can offer multiple language versions and culturally adapted interfaces to accommodate differences among African consumers in different regions while profiling consumers to understand the level of their acculturation. Respect for cultural differences is key to appropriately adjusting service and marketing strategies, thereby improving the impact of service quality on consumers’ perceived value.

### 6.3. Research Limitations and Future Works

In this study, there are some potential areas for improvement owing to the research conditions. Firstly, the sample of respondents consists primarily of undergraduate and graduate students from universities. This selection was made based on the expectation that this group possesses greater proficiency and interest in using e-commerce platforms. However, this choice has resulted in the homogeneity of the sample, which may limit the representativeness of the results for the broader consumer population. Therefore, it is recommended that future studies include a more diverse range of consumer groups beyond just students and examine the effects of demographic variables on the findings. Secondly, this study primarily focuses on the purchasing behavior of African consumers on CBEC platforms. Nevertheless, given that differences in cultural backgrounds can have significant impacts on consumer behavior, the generalizability of the study’s findings to other countries and regions may be limited. To address this, it is necessary to verify the study’s findings with other consumer groups in future studies. Additionally, this study mainly investigates the impact of acculturation on the relationship between CBEC platforms and consumers’ purchase intention. Going forward, it is recommended the moderating role of other related variables in this relationship is explored further.

## Figures and Tables

**Table 1:** Sample statistic distribution.

Item	Options	Frequency	Ratio	Item	Options	Frequency	Ratio
Age	Under 18 years old	4	0.96%	Level of education	Junior high school and below	1	0.24%
	18–30 years old	310	74.52%		High school	138	33.17%
	31–40 years old	92	22.12%		Junior college	40	9.62%
	41–50 years old	8	1.92%		Undergraduate	216	51.92%
	Over 50 years old	2	0.48%		Postgraduate	21	5.05%
Gender	Male	351	84.38%	CBEC shopping experience	Less than 1 year ago	207	49.76%
	Female	65	15.63%		1–3 years ago	109	26.20%
Annual personal disposable income	<$500	238	57.21%		3–5 years ago	48	11.54%
	$500–$1000	86	20.67%		5–10 years ago	25	6.01%
	$1000–$2000	52	12.50%		Over 10 years ago	27	6.49%
	>$2000	40	9.62%	Annual online shopping expenditures	<$100	262	62.98%
Overseas living experience	Never	330	79.33%		$100–$200	63	15.14%
	Less than 1 year ago	36	8.65%		$200–$300	36	8.65%
	1–3 years ago	30	7.21%		$300–$350	19	4.57%
	Over 3 years ago	20	4.81%		>$350	36	8.65%

**Table 2:** Conceptual measurement.

Variables	MI	Items	Reference
Information quality	IQ1	This CBEC platform provides correct information about the product/service.	[55]
	IQ2	This CBEC platform provides useful information about the product/service.	
	IQ3	This CBEC platform provides reliable information about the product/service.	
	IQ4	This CBEC platform provides sufficient information when I try to make a transaction.	
System quality	SYQ1	I use the system of this CBEC platform easily.	[61]
	SYQ2	The system of this CBEC platform equips with useful features and functions.	
	SYQ3	The system of this CBEC platform is friendly to me.	
	SYQ4	The system of this CBEC platform has a short response time for online processing.	
Service quality	SEQ1	The consumer service of this CBEC platform is willing to help me deal with problems.	[49,61]
	SEQ2	The consumer service of this CBEC platform provides me with individual services to meet my specific needs.	
	SEQ3	The logistics service provided by this CBEC platform is reliable and fast.	
	SEQ4	This CBEC platform supports multiple payment methods.	
	SEQ5	This CBEC platform gives prompt service to me.	
Acculturation	AC1	I care about the culture of the country to which this CBEC platform belongs.	[101,102]
	AC2	I use other websites/APPs of the country to which this CBEC platform belongs.	
	AC3	I often watch movies from the country to which this CBEC platform belongs.	
	AC4	I often listen to music from the country to which this CBEC platform belongs.	
Perceived value	PV1	The product/service of this CBEC platform is a good value for money.	[49]
	PV2	The price of this CBEC platform’s product/service is acceptable.	
	PV3	The product/service of this CBEC platform is considered to be a good buy.	
Consumer trust	CT1	I think this CBEC platform is trustworthy.	[103]
	CT2	This CBEC platform gives me the impression of keeping its promises and commitments.	
	CT3	I believe that this CBEC platform has my best interests in mind.	
Purchase intention	PI1	I am likely to purchase products on this CBEC platform.	[103,104]
	PI2	I am likely to recommend this CBEC platform to my friends.	
	PI3	I will probably make another purchase from this e-commerce platform if I need to buy cross-border products.	

**Table 3:** Results of reliability and convergent validity analysis.

Variables	AVE	CR	Cronbach’s Alpha	MI	Factor Loadings	T-Value
Information quality	0.717	0.91	0.868	IQ1	0.848	46.058
				IQ2	0.885	68.647
				IQ3	0.854	42.198
				IQ4	0.799	25.21
System quality	0.699	0.902	0.855	SYQ1	0.855	43.47
				SYQ2	0.888	66.227
				SYQ3	0.859	50.279
				SYQ4	0.733	19.879
Service quality	0.685	0.916	0.885	SEQ1	0.823	35.192
				SEQ2	0.841	35.336
				SEQ3	0.838	38.851
				SEQ4	0.806	31.457
				SEQ5	0.831	36.55
Acculturation	0.734	0.917	0.88	AC1	0.846	50.468
				AC2	0.867	51.898
				AC3	0.855	49.71
				AC4	0.859	45.707
Perceived value	0.859	0.948	0.918	PV1	0.923	96.433
				PV2	0.927	99.291
				PV3	0.931	112.126
Consumer trust	0.826	0.934	0.895	CT1	0.92	86.519
				CT2	0.924	84.542
				CT3	0.882	45.486
Purchase intention	0.863	0.95	0.92	PI1	0.918	55.278
				PI2	0.943	146.765
				PI3	0.925	82.215

**Table 4:** Results of discriminant validity analysis.

	1	2	3	4	5	6	7
Information quality	0.847						
System quality	0.726	0.836					
Service quality	0.713	0.78	0.828				
Acculturation	0.57	0.615	0.693	0.857			
Perceived value	0.741	0.77	0.808	0.779	0.927		
Consumer trust	0.664	0.715	0.748	0.695	0.822	0.909	
Purchase intention intention	0.676	0.739	0.735	0.626	0.798	0.812	0.929

Notes: Square root of AVE on diagonals.

## Data Availability

The main data and models generated or used during the study appear in the submitted article; the others are available from the corresponding author, on request.
